# Hail Lifestyle Medicine consensus position statement as a medical specialty: Middle Eastern perspective

**DOI:** 10.3389/fpubh.2025.1455871

**Published:** 2025-06-20

**Authors:** Saleh Alrajhi, Ayman Afify Konswa, Nisreen Alhamdi, Farhan Alshammari, Alaa Alqurashi, Rajaa Alraddadi, Hani Alfheeaid, Saad Albattal, Norah Alsoqih, Faisal Almuhaileb, Meshal Alnais, Edward Kunonga, Samia Latif, Sley Tanigawa Guimaraes, Rabbanie Tariq, Talal Albishri, Khalid Alrasadi

**Affiliations:** ^1^Research Center, King Fahad Medical City, Riyadh Second Health Cluster, Riyadh, Saudi Arabia; ^2^Prince Sultan Military Medical City, Riyadh, Saudi Arabia; ^3^Family Medicine, King Abdulaziz University, Jeddah, Makkah, Saudi Arabia; ^4^College of Nursing, University of Hail, Ha'il, Saudi Arabia; ^5^Gulf Health Council, Riyadh, Saudi Arabia; ^6^King Abdul Aziz University Hospital, Jeddah, Makkah, Saudi Arabia; ^7^Department of Food Science and Human Nutrition, College of Agriculture and Food, Qassim University, Buraydah, Saudi Arabia; ^8^National Institute of Health and Care Research, Applied Research Collaboration North East and North Cumbria, Newcastle, United Kingdom; ^9^UK Health Security Agency (UKHSA), London, United Kingdom; ^10^American College of Lifestyle Medicine (ACLM), Chesterfield, MO, United States; ^11^Preventive Medicine, Sher-I-Kashmir Institute of Medical Sciences, Srinagar, Jammu and Kashmir, India; ^12^College of Nursing, University of Qassim, Buraydah, Saudi Arabia; ^13^Medical Research Center, Sultan Qaboos University, Muscat, Oman

**Keywords:** Hail Lifestyle Medicine consensus, Lifestyle Medicine, Middle Eastern and North African countries (MENA), Saudi Arabia, LM conferencing model (SALM-CM^®^), digital AlAfiah system (DAS^®^)

## Abstract

**Background and importance:**

Lifestyle choices and practices are often the primary contributors to most preventable chronic diseases encountered in both outpatient and inpatient settings worldwide. Lifestyle medicine (LM) therapeutic interventions have consistently been shown through numerous scientific studies to improve and, in many cases, reverse chronic diseases. Globally, there is widespread acceptance of the 15 core competencies, 6 pillars, and the overarching definition of Lifestyle Medicine (LM) established by the American College of Lifestyle Medicine and its partners. While these 6 pillars provide a robust framework, they may not fully address the diverse needs of individuals and populations across various cultures, countries, and communities. The implementation of LM is inherently context-sensitive, influenced by factors such as local legislation, culinary traditions, food availability, economic conditions, agricultural development, whole-food retail accessibility, healthcare infrastructure, training opportunities, community resources, and faith or religious practices at both the individual and community levels. Furthermore, the MENA region, in particular, experiences a disproportionately high prevalence of lifestyle-related non-communicable diseases (NCDs) such as obesity, impaired glucose tolerance (IGT), type 2 diabetes mellitus (T2DM), hypertension (HTN), coronary artery disease (CAD), polycystic ovary syndrome (PCOS), and various forms of cancer, along with other related syndromes and co-morbid conditions. Addressing these challenges requires careful consideration of the local context—a complex interplay of culture, traditions, beliefs, and behaviors—that significantly shapes lifestyle choices, resource availability, and their subsequent impact on health and wellness outcomes. Significantly, the Hail Lifestyle Medicine International Conferences held in 2022 and 2023 have emphasized the strategic importance of Lifestyle Medicine in the MENA region, particularly in Saudi Arabia. These conferences underscored the need to define the scope of practice for Lifestyle Medicine in alignment with the ongoing Saudi healthcare transformation, the region's unique societal features, available local resources, and the specific needs of the population. Furthermore, these conferences served as pivotal platforms for convening experts, facilitating knowledge exchange, and fostering collaborations to tackle the distinct health challenges prevalent in the region. To provide a more comprehensive understanding, information regarding the establishment of the technical working group for the MENA region—critical to conceptualizing and adapting the LM pillars—should be included in the background or methods section earlier in the paper. This addition would offer readers insights into the foundational steps and collaborative efforts that initially shaped the initiative.

**Establishment of the technical working group for the MENA region:**

In response to the rising prevalence of non-communicable diseases (NCDs) and the pressing need for a tailored approach to Lifestyle Medicine (LM) in the MENA region, a Technical Working Group (TWG) was established. The initiative sought to adapt the six pillars of LM to align with the region's unique cultural, economic, and social contexts. The TWG comprised experts from various disciplines, including family medicine, preventive medicine, public health, nutrition, and related fields, ensuring diverse perspectives and expertise in primary care and lifestyle interventions. The concept of adopting expanded LM pillars was initially formulated during a series of international and regional conferences, notably the Hail Lifestyle Medicine International Conferences held in 2022 and 2023. These conferences highlighted the urgent need for a region-specific approach to mitigate the high prevalence of lifestyle-related NCDs in the MENA region. The TWG was tasked with conducting a comprehensive review of the existing LM pillars while taking into account critical factors such as local legislation, culinary traditions, food resources, economic conditions, and health systems infrastructure. To achieve expert consensus on the adapted LM pillars, the Delphi process was employed. This structured communication method involved several rounds of anonymous questionnaires, enabling experts to provide their input and refine their responses based on collective feedback. The process ensured a balanced, inclusive approach that minimized potential power imbalances and produced LM pillars tailored to the MENA region's specific needs.

**Methods:**

A survey was distributed to professional group panels representing multiple healthcare specialties, as well as to researchers and healthcare academic leaders of Lifestyle Medicine (LM) across Saudi Arabia and the broader Middle East. A Strength, Weakness, Opportunity, and Threat (SWOT) analysis was conducted, and the Delphi method was employed for structured communication and opinion formation. Using the nominal polling technique, formal responses were collected to develop an official written consensus position statement. The survey questions were validated and approved by an expert panel before being disseminated to the national Lifestyle Medicine group via WhatsApp for voting. Participants were asked to select one of three options: retain the American LM pillars, expand to the newly validated Saudi Arabian (MENA) LM pillars, or abstain from voting (neutral).

**Conclusions and relevance:**

Out of 815 members in the national Lifestyle Medicine (LM) group, 136 responses were received, while 679 members abstained from voting, indicating neutrality. Among the 136 votes, 36 supported retaining the LM pillars developed by the American College of Lifestyle Medicine AMCL and its partners without modifications. In contrast, 118 voted in favor of the newly developed MENA/Saudi Arabian pillars, acknowledging the need to address local healthcare needs that extend beyond traditional or classical medicine. The new pillars incorporated motor vehicle driving disturbances as a leading modifiable lifestyle factor contributing to death and disability in the region. Additionally, the impact of smart technology, particularly mobile phones and other devices, was recognized as a key contributor to motor vehicle accidents (MVAs), which significantly increase mortality and disability rates. Further, the new pillars emphasized emotional and mental health, spirituality, and sexuality, driven by the growing demand for comprehensive mental and emotional healthcare combined with faith-based and spiritual empowerment. Finaly, the inclusion of sexuality as a pillar was prompted by rapid societal changes, the rising prevalence of risky sexual behaviors, and the increased incidence of sexually transmitted infections (STIs). These challenges, coupled with the ambiguity surrounding who should manage such patients and the need for timely access to care, highlighted amajor gap in addressing this critical aspect of human health and quality of life. This addition ensures that LM physicians can provide appropriate care to close the gap in services related to sexual health.

## 1 Introduction

All healthcare professionals, particularly frontline consultants and trainees in family medicine, general pediatrics, preventive medicine, internal medicine, and OB-GYN, must understand the impact of evidence-based Lifestyle Medicine (LM) therapeutic interventions. These interventions influence genetics, epigenetics, and chronic disease management, ultimately affecting generational traits, including the potential emergence and prevalence of chronic diseases in future generations (1–5).

Approximately 80% of health-related conditions are attributed to factors beyond the control or scope of healthcare systems. For instance, public health policies and practices, access to healthy foods, physical activity empowerment, urban planning, housing, environmental pollution, social interaction norms and dynamics, faith-based local traditional practices in nutrition, human connectedness, emotional wellbeing, mental and social security, freedom of movement, water, sanitation, traffic calming and safety, substance use and abuse, and smart technology use and abuse (6, 7).

Lifestyle Medicine (LM) has been increasingly recognized as a global medical specialty. For example, the British Society of Lifestyle Medicine (BSLM), established in 2016, has played a pivotal role in advancing LM within the UK. The BSLM offers a diploma in Lifestyle Medicine, certified by the International Board of Lifestyle Medicine, and has incorporated LM into academic curricula at institutions such as Cambridge University (8).

In the United States, LM is also well-established, with numerous medical schools integrating it into their programs. Professional organizations like the American College of Lifestyle Medicine (ACLM) are at the forefront of these efforts. The ACLM offers certification for healthcare professionals and advocates for the widespread adoption of LM within mainstream healthcare (9, 10).

Globally, the Lifestyle Medicine Global Alliance (LMGA) connects regional members from around the world and provides online training to advance Lifestyle Medicine (LM). This alliance underscores the critical role of LM in addressing non-communicable diseases (NCDs) and improving overall health outcomes (9, 10).

These examples illustrate the increasing global acceptance and adaptation of LM as a recognized medical specialty. They highlight its transformative potential to enhance healthcare systems and improve patient outcomes.

In the MENA region, where Arabic is the most widely used official language, and Islam is the predominant religion, many shared values are accepted as societal norms across the population (11).^1^ These norms, combined with modern life and living practices, community and environmental interactions, nutritional choices, globalized economies, and the adoption of westernized meals and beverages, have contributed to a significant and exponential rise in the prevalence of preventable chronic diseases across all age groups, irrespective of economic status.

This statement was voted on by 815 members of the national Lifestyle Medicine (LM) group, who served as delegates at the Second Hail LM International Conference held in May 2023. Additional polling and voting were conducted through surveys of LM professional group panels comprising practicing healthcare specialists, researchers, and academic leaders from Saudi Arabia and other Middle Eastern countries. A SWOT analysis was performed, employing the Delphi method for communication and opinion formation. The nominal polling technique was used to collect opinions and formal votes.

The objective was to provide a MENA perspective on this emerging medical specialty to ensure a deeper and more effective application for the local population, including both healthcare practitioners and patients. This approach utilizes culturally adapted Lifestyle Medicine (LM) pillars designed to enhance the efficacy and efficiency of interventions for greater impact.

This initiative reflects and summarizes a cumulative effort spanning the last 6 years aimed at formulating the best medical practices for chronic disease management, improving quality of life and longevity, and reducing the disease burden and healthcare economic costs for the MENA population. Furthermore, this statement serves as an example of local contextualization, which could be extrapolated and adapted by other regions or countries worldwide.

## 2 Methods

To evaluate the degree of understanding, applicability, sensitivity, contextualization, and formalization of the newly adapted and expanded Lifestyle Medicine (LM) pillars and specialty in addressing the healthcare needs of Hail Province, Saudi Arabia, and the MENA region, a two-phase method was utilized.

Initially, the Hail Health Cluster initiatives were implemented as part of a broader effort to transform the healthcare sector in the Hail region in alignment with Saudi Arabia's Vision 2030 (12). These initiatives focus on several key areas:

1. **Disease Prevention**: Emphasizing lifestyle medicine, family medicine preventive services, and early disease detection to reduce and or reverse the incidence of pre-chronic diseases and chronic diseases.
2. **Improving Disease Management**: Enhancing the quality and safety of healthcare services to better manage existing conditions and improve patient outcomes.
3. **Shifting the Patient Pathway**: Encouraging the use of lifestyle medicine interventions in primary care centers as the first point of contact, thereby reducing the burden on hospitals and ensuring more efficient use of healthcare resources.

These initiatives aim to provide faster and more accessible healthcare services to all population segments, enhance population health through continuous assessment and targeted interventions, and measure outcomes to facilitate ongoing improvements.

### 2.1 Phase I Delphi exercise (drafting the questions and significance of the pillars)

The Hail conference was utilized as a platform to implement the Delphi exercise. The Delphi consensus process, employed by the panel, is a structured communication technique designed to achieve expert agreement. The panel consisted of expert leaders and local pioneers in the field, representing the Lifestyle Medicine National Group, the LM Scientific Foundation, and the LM Research Task Force.

The Delphi method involved multiple rounds of questionnaires, allowing experts to provide their opinions anonymously. After each round, a facilitator summarized the responses, and participants were encouraged to revise their answers based on the group's feedback. This iterative process continued until consensus was achieved or no further changes were noted (13–16).

In the first two rounds of the Delphi process, ideas and notes gathered from prior communications—including workshops, Zoom meetings, and social media interactions—were presented. These discussions, held among panel members between 2018 and 2023, served as a foundation for developing regionally adapted definitions and pillars. The nominal group technique was employed during these meetings to propose definitions and pillars that align with the region's cultural and religious context.

Additionally, a Key Care Indicator (KCI)^®^ was proposed as a valid measurement tool for evaluating Lifestyle Medicine outcomes in both community and medical clinic settings. By the end of the second round, the panel achieved an 80% consensus on the definitions presented.

The **last Delphi rounds** drafted the list of proposed questions and collected answers to compile a list of definitions.

The following questions have been proposed:

1. What are the unique features of dominant MENA cultures that influence lifestyle choices and practices that need to be addressed in a healthcare setting?
2. How can Lifestyle Medicine's definition, pillars, and core competencies be adapted, expanded, and built upon to meet local needs?
3. Who should receive training to practice lifestyle medicine, and why?
4. What are the research opportunities of LM in the MENA region?
4.1. National LM research initiatives
4.1.1. NCD continuous yearly mapping
4.1.2. NCD LM therapeutic intervention studies
4.1.3. NCD LM therapeutic intervention-pharmaceutical studies
4.1.4. NCD LM pillars validation studies
4.1.5. Mindset and mindfulness studies
4.1.6. Faith, customs, and health interaction
5. What is the Saudi Arabia, Hail Province, and Hail City experience?
5.1. Experience and outcome of LM scientific conferencing (20%), combined with public, private, community, individual, and family engagement (80%), i.e., (20+80) conferencing.
5.2. LM academic programs initiatives
5.3. LM Training of Trainers (TOT) initiative
5.4. LM speaker training initiative
5.5. LM CPG development initiative
5.6. Community, private, and public organizations engagement
6. What was learned from Saudi Arabia's LM conferencing model (SALM-CM), Hail's Pilot?
6.1. Science presentation to specialists and the public
6.2. Public research engagement
6.3. Community Physical Activity Engagement Initiatives
6.4. Community nutrition display, healthy food prep, and training initiatives
6.5. Community awareness programs
6.6. Public entities engagement initiatives
6.7. Eco-conferencing and environmental awareness initiatives

The expert panel members were predominantly family medicine consultants certified in Lifestyle Medicine (LM). The majority of the panel comprised practicing physicians (70%), followed by allied health professionals (20%) and researchers or academics (10%). With extensive experience in primary care, preventive medicine, and lifestyle interventions, the panel members brought diverse expertise across clinical practice, research, and teaching. This multidisciplinary background equipped them to provide valuable insights into the integration of LM into primary care.

To ensure the validity of the consensus, the panel assessed potential power imbalances among its members. This assessment included evaluating the panel's diversity in terms of geographical representation, professional roles, and years of experience. By addressing these factors, the panel aimed to minimize any potential influence of power imbalances on the consensus-making process.

### 2.2 Phase II online survey to external experts to vote for the proposed draft list

We used convenience sampling to identify social media platforms, selecting WhatsApp professional groups and professional email contacts from a database of 815 individuals. An online survey was distributed to these individuals, consisting of two main questions regarding the selection of either the American Lifestyle Medicine pillars or the newly developed MENA Lifestyle Medicine pillars (see Appendix A). Responses were recorded on a 5-point Likert scale.

Only 20% of respondents identified themselves as eligible to answer the questions, while the remaining 80% remained neutral, stating that they were not experts in Lifestyle Medicine.

Survey data were downloaded from Google Forms and analyzed using SPSS v.12. Descriptive analyses, including bar charts and percentages, were employed to describe the collected variables.

## 3 Results

### 3.1 Phase I

#### 3.1.1 Lifestyle medicine and related Saudi Arabia, MENA contextual definitions

For Saudi Arabia and MENA countries, the consensus panel has unanimously adopted Dr. Alrajhi's definition of Lifestyle medicine. It is best suited to meet current and future healthcare system transformation needs, published goals, and objectives. It puts LM at the heart of the Saudi Arabian Model of Care (MoC), where it is needed the most (Table 1). In addition, the group agreed on the newly adapted pillars of LM, which are presented in Table 2.

**Table 1:** Saudi Arabia, MENA contextual definitions.

**Term**	**Definitions**
Lifestyle medicine (LM)	LM is a form of total healthcare that employs AlAfiah^®^ evidence-based health care practices at individual and population levels. It utilizes preventive, pro-active, and pre-emptive strategies supporting timely access to healthcare services, cost effectiveness, healthy disease-free living, chronic disease reversal, and improving quality of life by employing the resources of public and private entities, communities, and individuals.
Alafiah^®^ medicine	AlAfiah^®^ is the sum of physical, mental, psychological, emotional, social, spiritual, environmental, economic health, universal living security, and wellbeing. It is influenced by genetics, epigenetics, education, faith, economic status, life and living security, nutrition, and environment. Moreover, it utilizes the shared resources of individuals, families, groups, societies, cultures, faiths, governments, and private and public entities. It is supported by an advanced and integrated AI, machine learning, and digital AlAfiah system (DAS^®^).
DAS^®^	AlAfiah Digital system, DAS^®^, under development, is an advanced system of healthcare, social, and human services that uses AI and Machine learning. It encompasses DAS EMR^®^, with integrated multi-stake holder capabilities (IMSH-C^®^).
Mindfulness (Ehsan)	Ehsan is being fully aware and conscious, or having super awareness and consciousness, in the pursuit of quality life and living, internal calm, peace, security, and justice. It is achieved through the mindset and intention of doing no harm, helping the individual to focus on beneficence and non-maleficence, with overall goals to be beneficial to one's self, family, community, and humanity. For example, focusing life and living activities on positive, productive, and psychologically elevating activities, having stronger family, neighbors, and friends ties, eating/sleeping/playing better, and optimizing faith and spirituality. This is done through evidence-based learning, knowledge acquisition, and continuous life and living improvement.
Key care indicator (KCI)^®^	KCI^®^ is a chronic disease quality of care indicator that measures holistic medical management outcome, with a focus on disease remission, improvement, reduction of co-morbidities, disease/economic/social/and psychological burden, quality of life and living, wellbeing, and pharmaceutical burden, to mention some. KCI^®^ scope
Mindset, the power of (Aleman and AlFikr)	The Arabic word Aleman represents a wider sense of what a Mindset is. Further, the Arabic definition is broader and adds faith and evidence-based beliefs that enhance internal, spiritual, psychosocial, emotional, and physical resilience and wellbeing, i.e., Alafiah^®^. In cognitive psychology, a mindset is the cognitive process activated in a task (6).
Last, new SALM-CM^®^ conferencing model	SALM-CM has been developed to engage all healthcare providers, regulators, government and private organizations and authorities, individuals, families, commercial entities, and the community. This model has been refined twice over a period of 5 days each in Hail province, Saudi Arabia. Each conference has an executive plan covering the following topics, functions, and activities: 1- SALM-CM^®^ is a pilot for professional, public, and private partnership and collaborative empowerment 2- Scientific program of 20–30 CME/CE hours (for healthcare professionals, researchers, academicians, and industry leaders) 3- Emphasis on Indoor and Outdoor conferencing 4- Research session (covering all research related to LM), with public engagement display and session 5- Eco-conferences (reforestation of the desert as a part of Green Saudi Arabia projects) emphasizing the importance of healthy outdoors, environmental care, and sustainable living 6- Sports and Physical activity competitions 7- Healthy cooking competitions (inter-provincial and local) 8- Partnership and engagement with government ministries, authorities, agencies 9- Partnership and engagement with chamber of commerce, private companies, food produces, and sellers 10- Community-wide outreach with local leaders, influencers, and the general public through joint physical activity and health nutrition initiatives, awareness lectures, and programs 11- Educational institutions and universities' partnership 12- LM education, higher education, and training opportunities initiatives 13- Whole food and plant food business opportunities initiatives 14- Introduction of KCI^®^ as a measure of LM therapeutic effectiveness 15- Introduction of Alafiah Medicine

**Table 2:** The newly adapted LM pillars to the Middle Eastern population according to the task force analysis.

**Adapted LM pillars to the Middle Eastern population**
1. Food as medicine
1.1 Generational management (pre-conception to older adults)
2. Physical activity and exercise as medicine
3. Sleep health and sleep disorders
4. Life, living, and stress management
4.1. Social connectedness (Alselah and Alber)
4.2. Mindset, the power of (Aleman and Fikr)
4.3. Mindfulness (Alehsan)
4.4. Work burnout
4.5. Community and social determinants of health (cultural ethics and moral norms)
5. Addiction and Substance Abuse Management
6. Driving disturbance
7. Smart technology disturbance
8. Spirituality
9. Mental and emotional health
10. Sexuality health

### 3.2 Phase II

Out of 815 participants who received the survey, 136 completed the questions, while the remaining 679 participants chose to remain neutral and abstained from voting, citing a lack of adequate experience or certification in Lifestyle Medicine.

**Figure 1:**
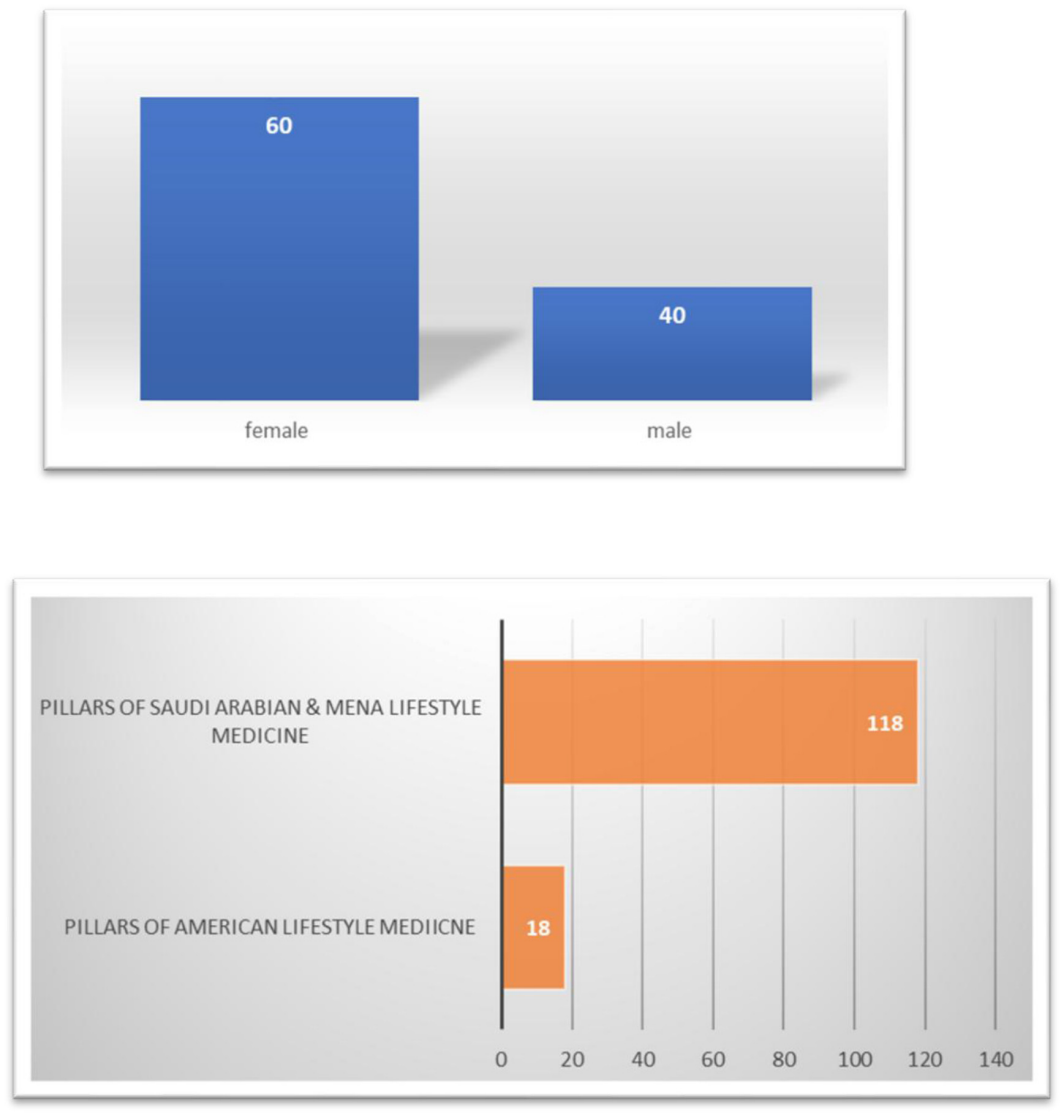
Response categories and characteristics.

Among the 136 respondents, 118 professionals voted in favor of adopting the newly adapted pillars tailored for Saudi Arabia and the MENA region, whereas 18 professionals opted to retain the American Lifestyle Medicine pillars (Figure 1). The majority of respondents were female (60%), with the remaining 40% being male. Regarding occupations, 70% of participants were physicians, followed by allied health professionals (20%) and researchers or academics (10%).

More than half of the professionals work within healthcare systems, providing direct Lifestyle Medicine (LM) care alongside their primary specialties. The majority of participants (80%) work and live in Saudi Arabia, while a smaller proportion (20%) are from the broader MENA region. The common age range among respondents was 30 to 60 years, with an average age of 45 years.

The neutrality of the 679 participants underscores the importance of having sufficient experience and certification in Lifestyle Medicine to make informed decisions. It also highlights the need for ongoing education and training in this field, ensuring that more healthcare professionals feel confident participating in similar surveys in the future.

## 4 Discussion

The interplay between Lifestyle Medicine and mental and emotional health is complex and multifaceted. It involves the Social Determinants of Health (SDH) and lifestyle, which are increasingly recognized as critical factors in predicting health outcomes for both populations and individuals. These determinants include the conditions into which people are born, grow, work, live, and age, as well as the broader forces and systems shaping the conditions of daily life. Thus, an individual's lifestyle is inherently intertwined with SDH. Research suggests that up to 80% of chronic diseases and premature deaths could be prevented by avoiding smoking, engaging in regular physical activity, and adhering to a healthy diet (17). Moreover, a healthy lifestyle is positively associated with mental health and wellbeing, reducing the risk of developing mental health conditions or worsening existing ones.

The MENA region faces an increasing burden of lifestyle-related chronic diseases, including obesity, impaired glucose tolerance (IGT), type 2 diabetes mellitus (T2DM), hypertension (HTN), coronary artery disease (CAD), various cancers, and ~242 co-morbid conditions linked to adiposity. This challenge is compounded by inadequate public health policies, high tobacco use, sedentary lifestyles, sleep disorders, and limited access to whole and plant-based foods. Additionally, the widespread consumption of refined and ultra-refined foods, the Standard American Diet (SAD), and processed pre-prepared meals, drinks, snacks, and food items exacerbates the situation (18).

The populations in MENA countries also have unique, faith-oriented factors influencing their lifestyle choices and daily practices. These factors include food preferences, meal frequency, social relationships, physical activity, and decision-making. About 90% of the MENA population shares a common faith, which plays a pivotal role in the lifestyle determinants of health. Faith serves as a core cultural value for both medical staff and patients, guiding concepts such as *EMAN* (mindset) and *EHSAN* [mindfulness; (19–21)]. When these concepts are incorporated into medical consultations, they significantly enhance patient acceptance and compliance with chronic disease management.

The region is undergoing an international transformation toward modernization and technology adoption, leading to Westernized food habits and increasingly sedentary lifestyles. Over the past 30–50 years, non-communicable diseases (NCDs) have risen sharply due to lifestyle and environmental changes (22–24), placing an unsustainable demand on healthcare systems globally. This surge in NCDs, including cardiovascular diseases, cancers, respiratory diseases, and diabetes, has escalated healthcare costs and created significant resource allocation challenges, emphasizing the urgent need for effective prevention and management strategies.

In response, the Saudi National Lifestyle Medicine Group has developed an adaptation of the six pillars of Lifestyle Medicine, as originally defined by the American College of Lifestyle Medicine. This initiative, which began in 2018 and concluded in 2023, resulted in a consensus statement from delegates representing various Arab countries, with the majority of participants residing and working in Saudi Arabia.

A local expert panel, comprising professionals from diverse healthcare specialties, researchers, and academic leaders, formulated a consensus opinion defining the scope and practice of Lifestyle Medicine from both a MENA and Saudi Arabian perspective. This work aligns with the ongoing healthcare transformation in the region, addressing unique societal features, available local resources, and the specific needs of the population. The panel considered the interplay of local factors influencing illness and wellness, including the role of faith, beliefs, food choices, and social determinants of health.

This statement introduces contextualized Lifestyle Medicine (LM) pillars tailored for the Arab and MENA populations, integrating cultural and historical contexts, ethical and moral foundations, mental health, and self-satisfaction. It incorporates new concepts such as Eman and Fikr as fundamental components of mindset.

The definition of mindset within this framework includes beliefs, values, social and marital principles, financial dealings, and an individual's outlook on life, all shaped by personal experiences and acquired knowledge (19).

The MENA contextual definition of mindset refers to an established set of beliefs, concepts, doctrines, and attitudes shaped by experience and/or knowledge. It applies to individuals, families, communities, tribes, nations, or faith-based homogeneous groups and encompasses perspectives on morality, generational and global relationships, history, virtue, culture, social and marital principles, financial and economic dealings, values, and life principles.

Additionally, this mindset definition includes interpretations of justice, human rights, civil rights, hierarchies, governance, leadership, meaning of life, frame of mind, worldview, judgment principles, and overall disposition.

The collective components of a mindset drive decision-making, actions, reactions, acceptance and rejection, motivation, effort, allegiance, change, creativity, adaptation, and innovation in navigating life's continuum. It serves as the reference framework against which all aspects of life are evaluated for approval, adoption, rejection, disapproval, action, or reaction. Mindset is a complex set of cognitive processes that individuals use to arrive at justified, actionable decisions with minimal regrets (19, 21).

Additionally, the concept of *EHSAN* (mindfulness) emphasizes total effort and the pursuit of optimal outcomes, promoting better sleep, healthier eating habits, stress reduction, productivity, leisure, and physical activity. New pillars also address driving disturbances and the misuse of smart technology, acknowledging their significant impact on health.

Integrating spirituality into Lifestyle Medicine can enhance health outcomes by fostering wellness, increasing satisfaction with care, and improving medical decision-making (25). Spirituality and Lifestyle Medicine are interconnected in multiple ways. Research demonstrates a strong association between medicine, faith, religion, and spirituality. Spirituality is defined as the way individuals and groups seek and express meaning and purpose in life, experiencing social and faith-based connectedness with themselves, others, and the Almighty. It significantly influences wellness, satisfaction with care, medical decision-making, and overall health outcomes.

Despite its significance, the healthcare industry largely overlooks the role of faith and spirituality in wellbeing and illness. Research indicates that participation in faith-based religious communities is associated with healthier lives, greater longevity, lower rates of depression, reduced suicidal tendencies, and minimal to no substance abuse. Thus, integrating spirituality into Lifestyle Medicine pillars has the potential to enhance health outcomes and patient care (26).

Additionally, new concepts such as B'err and S'lah have been adopted to reflect the MENA region's strong emphasis on social connectedness, family ties, and community cohesion.

Furthermore, Lifestyle Medicine, sexuality, sexually transmitted infections (STIs), sexual behavior, and human connectedness are deeply interconnected. Sexuality is fundamental to human life, influencing healthy relationships from early adulthood through old age. Risky sexual behaviors can increase the likelihood of STIs, underscoring the need for preventive strategies within Lifestyle Medicine. This discipline seeks to understand the physical and psychological factors contributing to sexual health concerns. Unhealthy lifestyle choices—such as obesity, tobacco smoking, alcohol and substance abuse, and chronic stress—can lead to sexual dysfunctions, further highlighting the importance of integrating Lifestyle Medicine principles into sexual health management.

Human connectedness plays a crucial role in sexual health. Healthy relationships contribute to planned pregnancies and disease prevention, while sexual dysfunction may serve as an early indicator of future non-communicable diseases (NCDs), such as cardiovascular and metabolic disorders. Therefore, promoting healthy lifestyles is essential for ensuring a satisfying sexual quality of life (27).

Additionally, driving disturbances and smart technology disturbances were introduced as additional Lifestyle Medicine pillars. Maladaptive motor vehicle driving, which leads to serious car accidents, is the leading cause of death in Saudi Arabia. Similarly, the maladaptive use and abuse of smart technology have been identified as major contributors to sleep disturbances, mental and psychosocial disorders, obesity, and co-morbid diseases.

The phase two statement had several limitations, including its reliance on web-based surveys and a cross-sectional study design. The online platform restricted participation to individuals with internet access and those known to the statement group. Nonetheless, the statement's unique structure and its representation of the region remain significant.

## 5 Policymakers and lifestyle academy recommendation

A consensus opinion was formulated stating the following:

1. Maintaining and building upon the 6 pillars of commonly accepted lifestyle medicine specialty
2. Adapting the 6 pillars in number 1 to serve the local population, healthcare resources, and application needs.
3. Adding new pillars or sub-pillars to cover lifestyle-rooted causes of morbidity and mortality unique to the MENA, Saudi Arabia, region/country.
4. Expanding some pillars to suit local needs
5. Employing the full potential of LM for professional development, stakeholders, and community engagement.

## 6 Research recommendation or thoughts

### 6.1 Points for reflection and food for thought

Can the Saudi healthcare system adopt preventive, proactive, and pre-emptive care alongside wellness-focused initiatives, leveraging the strength of its infrastructure? Is there a determined commitment to positioning Saudi Arabia as a global leader in proactive health and wellness care? Furthermore, is there a formal strategy to incentivize wellness and sustain it effectively? Could the development of a medically driven reward system motivate individuals and contribute to achieving Alafiah?

Does Saudi Arabia have any way to re-distinguish itself, as it did with vaccinations?

## 7 Conclusions

The LM pillars have been expanded to address the professional and community needs in Saudi Arabia, serving as a model for other MENA countries and societies.

Rather than focusing solely on stress management as a contributor to ill health, a broader pillar, Life and Living Management, has been introduced. This expanded pillar encompasses stress, social and work interpersonal interactions, interdependence, work-related and social stress, as well as life and living resource management.

Additionally, concepts such as mindset, mindfulness, and social connectedness, which are deeply embedded in local faith teachings and practices, have been redefined and expanded to reflect local orientations. These elements have been integrated as highly effective tools to deliver care plans, facilitate life and living management, provide resilience training, and enhance compliance with LM therapeutic interventions.

## Data Availability

The original contributions presented in the study are included in the article/supplementary material, further inquiries can be directed to the corresponding author.

## Footnotes

^1^ https://www.islamicity.org/6546/ihsan-the-pursuit-of-excellence/

## Appendix A

### Survey questions

1-Do you think that the 6 pillars of lifestyle medicine are sufficient for the needs of Saudi Arabia and MENA region's population?

Whole Food, Plant-based Nutrition

Physical Activity

Stress Management

Avoidance of Risky Substances

Restorative Sleep

Social Connection

Strongly agree: Agree: Neutral: Disagree: Strongly disagree:

2-Do you think that the 6 pillars of lifestyle medicine's practice and principles can be adapted, expanded, and customized to meet regional and local population healthy living needs?

MENA Saudi Arabia Lifestyle Medicine Pillars adapted and expanded from ACLM pillars

1. Food as medicine

1.1. Generational management (pre-conception to older adults)

2. Physical activity as medicine
3. Sleep health
4. Life and Chronic Stress Management

4.1 Social connectedness (Alselah and Alber)
4.2 Mindset, the power of (Aleman and Fikr)
4.3 Mindfulness (Alehsan)
4.4 Work burnout
4.5 Community and social determinants of health (cultural ethics and moral norms)

5. Addiction and Substance Abuse Management

5.1 Tobacco use
5.2 Alcohol use
5.3 Illegal drugs use

6. Driving disturbance

6.1 MVA reduction and prevention

7. Smart technology disturbance
8. Sexuality health

Strongly agree: Agree: Neutral: Disagree: Strongly disagree:
